# Identification of the specific long-noncoding RNAs involved in night-break mediated flowering retardation in *Chenopodium quinoa*

**DOI:** 10.1186/s12864-021-07605-2

**Published:** 2021-04-19

**Authors:** Qi Wu, Yiming Luo, Xiaoyong Wu, Xue Bai, Xueling Ye, Changying Liu, Yan Wan, Dabing Xiang, Qiang Li, Liang Zou, Gang Zhao

**Affiliations:** Key Laboratory of Coarse Cereal Processing, Ministry of Agriculture and Rural Affairs, Sichuan Engineering & Technology Research Center of Coarse Cereal Industralization, School of Food and Biological Engineering, Chengdu University, Chengluo road 2025, Shiling town, Longquanyi District, Chengdu, 610106 Sichuan Province P.R. China

**Keywords:** LncRNA, Night break, Flowering, *Chenopodium quinoa*, Co-expression network

## Abstract

**Background:**

Night-break (NB) has been proven to repress flowering of short-day plants (SDPs). Long-noncoding RNAs (lncRNAs) play key roles in plant flowering. However, investigation of the relationship between lncRNAs and NB responses is still limited, especially in *Chenopodium quinoa*, an important short-day coarse cereal.

**Results:**

In this study, we performed strand-specific RNA-seq of leaf samples collected from quinoa seedlings treated by SD and NB. A total of 4914 high-confidence lncRNAs were identified, out of which 91 lncRNAs showed specific responses to SD and NB. Based on the expression profiles, we identified 17 positive- and 7 negative-flowering lncRNAs. Co-expression network analysis indicated that 1653 mRNAs were the common targets of both types of flowering lncRNAs. By mapping these targets to the known flowering pathways in model plants, we found some pivotal flowering homologs, including 2 florigen encoding genes (*FT* (*FLOWERING LOCUS T*) and *TSF* (*TWIN SISTER of FT*) homologs), 3 circadian clock related genes (*EARLY FLOWERING 3* (*ELF3*), *LATE ELONGATED HYPOCOTYL* (*LHY*) and *ELONGATED HYPOCOTYL 5* (*HY5*) homologs), 2 photoreceptor genes (*PHYTOCHROME A* (*PHYA*) and *CRYPTOCHROME1* (*CRY1*) homologs), 1 B-BOX type *CONSTANS* (*CO*) homolog and 1 *RELATED TO ABI3/VP1* (*RAV1*) homolog, were specifically affected by NB and competed by the positive and negative-flowering lncRNAs. We speculated that these potential flowering lncRNAs may mediate quinoa NB responses by modifying the expression of the floral homologous genes.

**Conclusions:**

Together, the findings in this study will deepen our understanding of the roles of lncRNAs in NB responses, and provide valuable information for functional characterization in future.

**Supplementary Information:**

The online version contains supplementary material available at 10.1186/s12864-021-07605-2.

## Background

Plants sense environmental changes to maximize flowering transition success. Accumulating evidences have revealed that six major factors, including temperature, photoperiod, gibberellin acids (GAs), age and autonomous pathways are implicated in flowering regulation [1]. The photoperiod pathway controls floral transition through a signal cascade involving circadian clock and florigen genes. Under inductive photoperiods when the expression phases of endogenous circadian clock regulators coincide with external light signal, the clock output genes are converged to activate the florigen-encoding genes, *FLOWERING LOCUS T* (*FT*) and its homologs [2–4]. Thereafter, FT proteins are transported to shoot apical meristems where interact with FLOWERING LOCUS T (FD) and 14–3-3 proteins to form florigen activation complex (FAC), which in turn induces the meristem identity gene *APETALA1* and triggers flowering [5, 6]. Day length is a critical factor for photoperiodic flowering. In short-day plants (SDPs) such as rice (*Oryza saltiva*) and soybean (*Glycine max*), *FT* transcripts are more abundant when the night length longer than a certain threshold [7, 8]. By contrast, in long-day plants (LDPs) such as Arabidopsis (*Arabidopsis thaliana*) and wheat (*Triticum aestivum*), only when the day length exceeds a certain threshold, FT proteins are sufficient for flowering induction [9, 10]. Different photoperiods combined with short pulses of light during the night period (referred to as night break, NB) approaches are well-established and widely adopted to study the photoperiodic regulation of flowering in many plant species. NB causes different effects in flowering of SDPs and LDPs by changing the expression of *FT*. In rice, *Heading date 3a* (*Hd3a*), a homolog of Arabidopsis *FT*, is the principle mediator responsible for NB flowering retardation [11]. In soybean, down-regulation of *FT2a* and *FT5a* is the major cause for NB responses [12]. In contrast, multiple NBs accelerate heading in wheat plants grown under SD by inducing *FT1* [13]. Genetic studies indicate that the transcriptional changes of *FT* in response to NB are predominantly mediated by phytochromes. NB responses are abolished in the *phyB* mutants of rice [14], *phyA* mutants of soybean [12] and the *phyB* and *phyC* mutants of wheat [13], indicating functional conservation and divergence of phytochromes across different plant species. Further studies indicate circadian clock genes are also involved in NB responses. The up-regulation of *FT1* by NB in wheat is dependent on *PHOTOPERIOD1* (*PPD1*), a homolog of *PSEUDO RESPONSE REGULATOR 37* (*PRR37*) in rice, which is a core component of the circadian clock [13]. In poplar (*Populus* L.), NB induces *FT2* to free the shoot growth cessation by suppressing the circadian clock gene *LATE ELONGATED HYPOCOTYL 2* (*LHY2*) [15]. Thus, NB affects plant flowering by controlling different layers of regulators.

Long-noncoding RNAs (lncRNAs) refer to a class of RNA transcripts longer than 200 nt which lack discernable protein coding potential [16, 17]. Compared with mRNAs, lncRNAs have much lower expression levels and sequence conservation [16, 17]. Most lncRNAs harbor spatio-temporal specificity [16, 17]. lncRNAs could be generated from the intergenic, intronic, or coding regions in the sense and antisense directions of mRNA [16, 17]. lncRNAs control the expression of target genes in *cis*- or *trans*-acting way [18, 19]. lncRNAs exert their functions by serving as sponge for miRNAs, functioning as precursors of miRNAs in the cytoplasm [20], or serving as scaffolds of epigenetic regulators to modulate chromatin status in the nucleus [17, 21]. A few studies indicate that lncRNAs participate in different biological processes, such as flowering, fertility, phosphate metabolism, leaf patterning, nodule formation and phytohormone-related development. *COLD INDUCED LONG ANTISENSE INTRAGENIC RNA*s (*COOLAIR*) and *COLD ASSISTED INTRONIC NONCODING RNA* (*COLDAIR*), two different classes of lncRNAs transcribed from the site of *FLOWERING LOCUS C* (*FLC*) in the antisense direction and from the first intron of *FLC* in the sense direction, respectively, are involved in flowering regulation by repressing *FLC* via epigenetic modulation [22–24]. *LD-SPECIFIC MALE-FERTILITY-ASSOCIATED RNA* (*LDMAR*) controls the photoperiod-sensitive male sterility in rice under LD [25]. The rice lncRNA *INDUCED BY PHOSPHATE STARVATION 1* (*IPS1*) functions as a target mimic of *miR399* to up-regulate the phosphate metabolism gene *PHOSPHATE2* (*PHO2*) when encountered with phosphate starvation [26]. *TWISTED LEAF* (*TL*), an antisense lncRNA of *OsMYB60*, suppresses *OsMYB60* via epigenetic chromatin modification to regulate leaf morphology [27]. In *Medicago truncatula*, the lncRNA *EARLY NODULIN 40* (*ENOD40*) *ENOD40* interacts with RNA-BINDING PROTEIN 1 (MtRBP1) to change its location from nuclear speckles to cytoplasmic granules to promote nodule formation [28]. The lincRNA *AUXIN REGULATED PROMOTER LOOP* (*APOLO*) regulates the promotor chromatin loop of *PINOID* (*PID*) which regulates polar auxin transport [29]. Recently, genome-wide identification of lncRNAs has been performed in a few plants. Huang et al identified 14 lncRNAs co-expressed with 10 pollen-associated coding genes during pollen development in *Brassica rapa* (*B. rapa*) [30]. One hundred forty-two lncRNAs may participate in fruit ripening and the climacteric in *Cucumis melo* by regulating the targets involved in auxin signal transduction, ethylene, sucrose biosynthesis and signaling and the ABA signaling pathway [31]. One hundred eighty-five salt stress-related lncRNAs were obtained in duckweed (*Spirodela polyrhiza*) [32]. By co-expression analysis, the important lncRNA-mRNA pairs associated with contrasting drought stress responses in two rapeseed cultivators (*B. napus*) were obtained [33]. These studies have shed lights on the roles of lncRNAs. However, the roles of lncRNAs in flowering regulation remain largely unknown, especially in quinoa (*Chenopodium quinoa*), an important SD coarse cereal.

Quinoa has been recommended by FAO as “super food” because of the nutritious elements in the seeds [34]. As quinoa is a facultative SD plant [35, 36], many photoperiod-sensitive quinoa accessions may encounter with yield penalties when grown across different latitudes. However, for the time being the lncRNAs and corresponding regulatory mechanisms involved in quinoa photoperiodic flowering remain to be elucidated. To this end, in the present study, we investigated the effect of NB on quinoa flowering, and by performing strand-specific RNA sequencing (SS RNA-seq) of the plants grown under NB and SD, we identified the specific coding genes and lncRNAs that may be involved in flowering regulation. Hence, this study provides novel insights into the quinoa flowering molecular mechanisms.

## Results

### Effects of short-day and night-break on quinoa flowering time

**Fig. 1:**
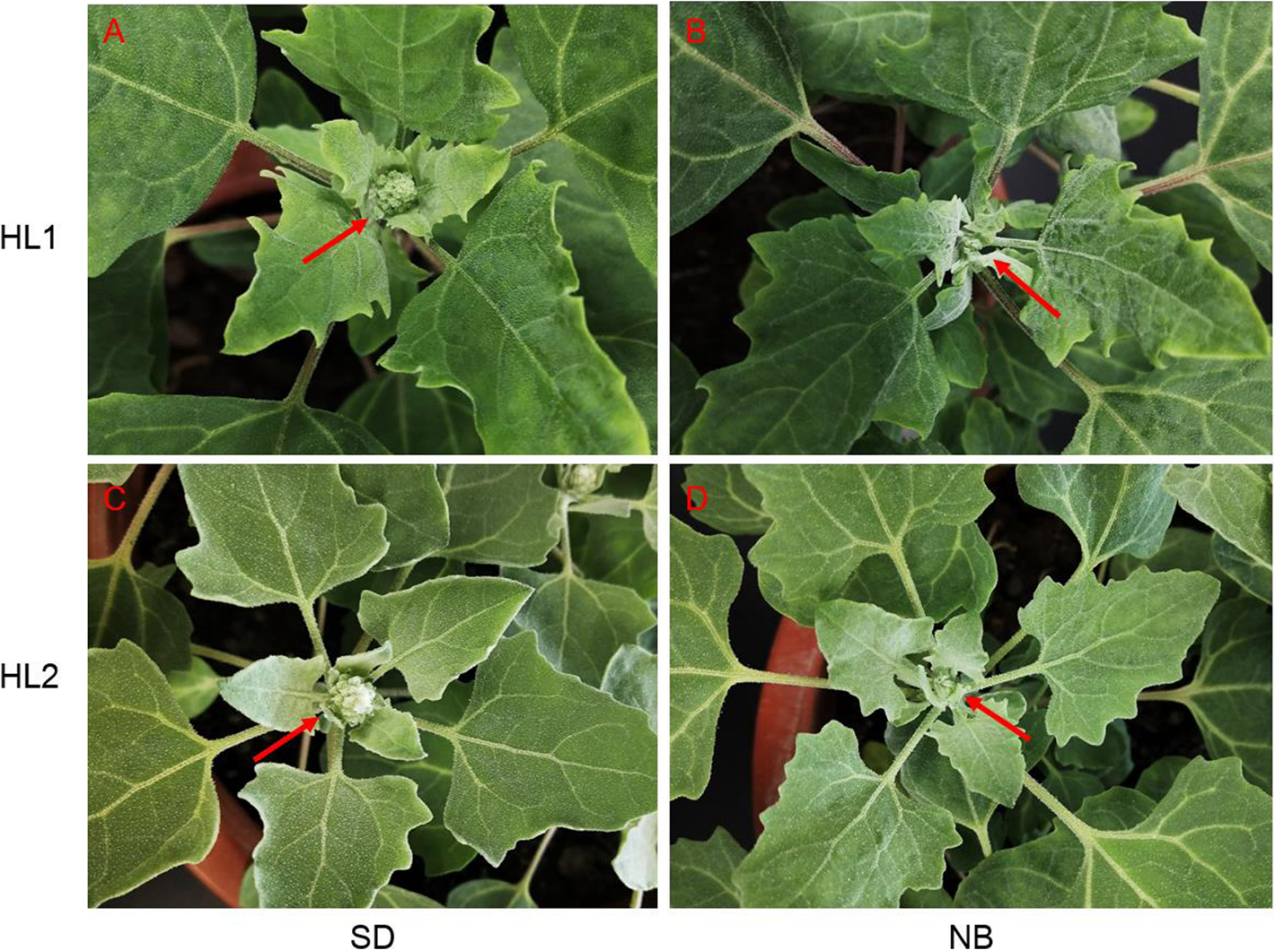
Phenotypes of the floral buds in two quinoa cultivars under SD and NB. On 40 DAS, the floral buds were apparently visible in cultivars “HL1” (**a**) and “HL2” (**c**) under SD, whereas under NB, the floral buds were very tiny in cultivars “HL1” (**b**) and “HL2” (**d**)

To evaluate the effects of short-day (SD) and night-break (NB) on quinoa flowering, two quinoa cultivars (“HL1” and “HL2”) with different maturation periods were used. Seeds were sown in pots and grown under natural day conditions before photoperiodic treatment. Then, 14 d-old seedlings were transferred into growth chambers and subjected to SD and NB treatment, respectively. After 14 d photoperiodic entertainment in growth chambers (28 d-after-sowing, 28 DAS), the floral buds initiated in “HL1” and “HL2” under SD, whereas no visible floral buds found in both cultivars under NB. On 40 DAS, the floral buds size was obviously bigger under SD (Fig. 1a, c) than that under NB (Fig. 1b, d). Thus, we speculated that SD accelerates whereas NB represses floral buds development.

### Transcriptome sequencing and identification of lncRNAs in quinoa

**Fig. 2:**
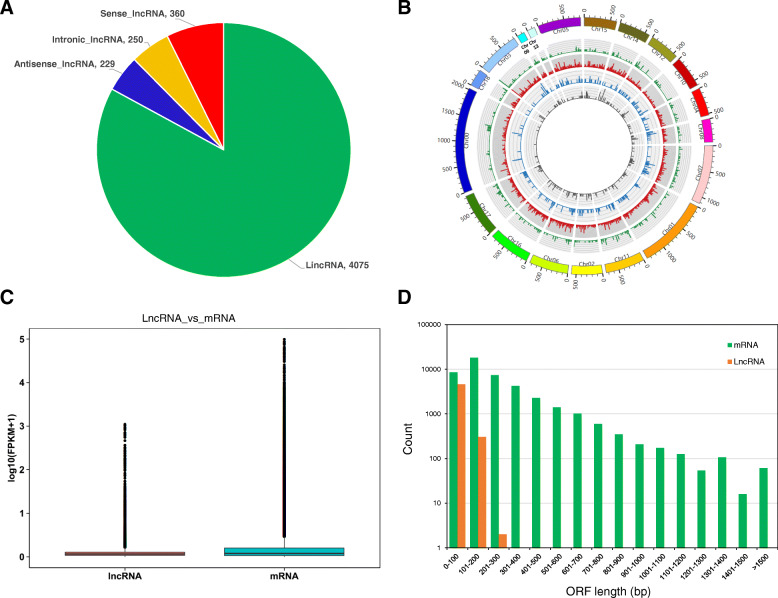
Characteristics of the short-day and night-break responsive lncRNAs identified from quinoa leaves. **a** Numbers of four types of lncRNAs. **b** Distribution of different-type lncRNAs on the quinoa chromosomes. Five circles from outside to inside represents quinoa chromosomes, sense lncRNAs (green), intergenic lncRNAs (red), intron lncRNAs (blue), antisense lncRNAs (gray). **c** Comparison of the expression levels of lncRNAs and mRNAs. **d** Open reading frame (ORF) length distributions of lncRNA and mRNA transcripts

To uncover the roles of lncRNAs in NB responses of quinoa, seedlings of “HL1” were grown in growth chamber and treated by SD and NB as described in section Methods. The leaf samples of “HL1” were collected on 14 DAS (SD1), 26 DAS (SD2) under SD, and 28 DAS under NB. Sampling was performed with three biological replicates for each time point. Then the samples were subjected to total RNA extraction and transcriptome analysis. Through SS RNA-seq on an Illumina Hiseq4000 platform, an average of more than 16.02 GB data of 9 cDNA libraries were obtained (Table 1). The Q30 value was more than 90.79% for each library (Table 1). Subsequently, the clean reads were mapped to the quinoa reference genome [37] using HISAT2 software [38], and the mapping rate was higher than 85.48% for each library (Table 1). Based on the mapping results, StringTie [39] was used to re-construct the transcripts and predict the novel genes. The assembled transcripts were annotated by Gffcompare program [40], and the unannotated transcripts were further used for long-noncoding RNA (lncRNA) prediction. Based on the criteria described in section Methods, a total of 4914 lncRNAs were identified. According to their genomic origins, 4075 intergenic lncRNAs (lincRNAs), 250 intronic lncRNA, 360 sense lncRNAs, and 229 antisense lncRNAs were identified (Fig. 2a), respectively. By performing BLAST on lncRNAs database, we found only 605 lncRNAs were annotated in the NONCODE database [41], and most (87%) of the quinoa lncRNAs were novel genes. Meanwhile, as shown in Fig. 2b, various types of lncRNAs were evenly distributed over the whole quinoa genome at the density of 3.5 per Mb.

**Table 1:** Summary of the clean reads obtained from 9 strand-specific cDNA libraries

Sample	Clean reads	Clean bases	GC Content	Q30	Mapped Reads	Unique Mapped Reads	Multiple Map Reads
SD1–1	120,575,592	18,024,639,138	42.06%	93.79%	105,438,405 (87.45%)	77,322,246 (64.13%)	28,116,159 (23.32%)
SD1–2	118,844,120	17,742,199,126	42.33%	94.07%	103,599,037 (87.17%)	75,650,640 (63.66%)	27,948,397 (23.52%)
SD1–3	109,416,192	16,342,140,838	42.23%	93.34%	94,815,186 (86.66%)	69,895,804 (63.88%)	24,919,382 (22.77%)
SD2–1	118,152,040	17,646,198,882	42.35%	93.17%	104,853,815 (88.74%)	72,776,293 (61.60%)	32,077,522 (27.15%)
SD2–2	156,229,454	23,239,577,248	42.31%	93.38%	138,496,418 (88.65%)	96,469,015 (61.75%)	42,027,403 (26.90%)
SD2–3	107,354,748	16,024,541,498	42.52%	90.79%	91,987,548 (85.69%)	64,905,652 (60.46%)	27,081,896 (25.23%)
NB-1	107,521,308	16,067,918,002	42.84%	91.25%	91,911,056 (85.48%)	67,788,130 (63.05%)	24,122,926 (22.44%)
NB-2	119,435,422	17,792,985,836	42.53%	93.07%	104,464,425 (87.47%)	76,143,854 (63.75%)	28,320,571 (23.71%)
NB-3	117,442,040	17,505,052,862	42.31%	94.01%	103,617,559 (88.23%)	75,619,123 (64.39%)	27,998,436 (23.84%)

**Fig. 3:**
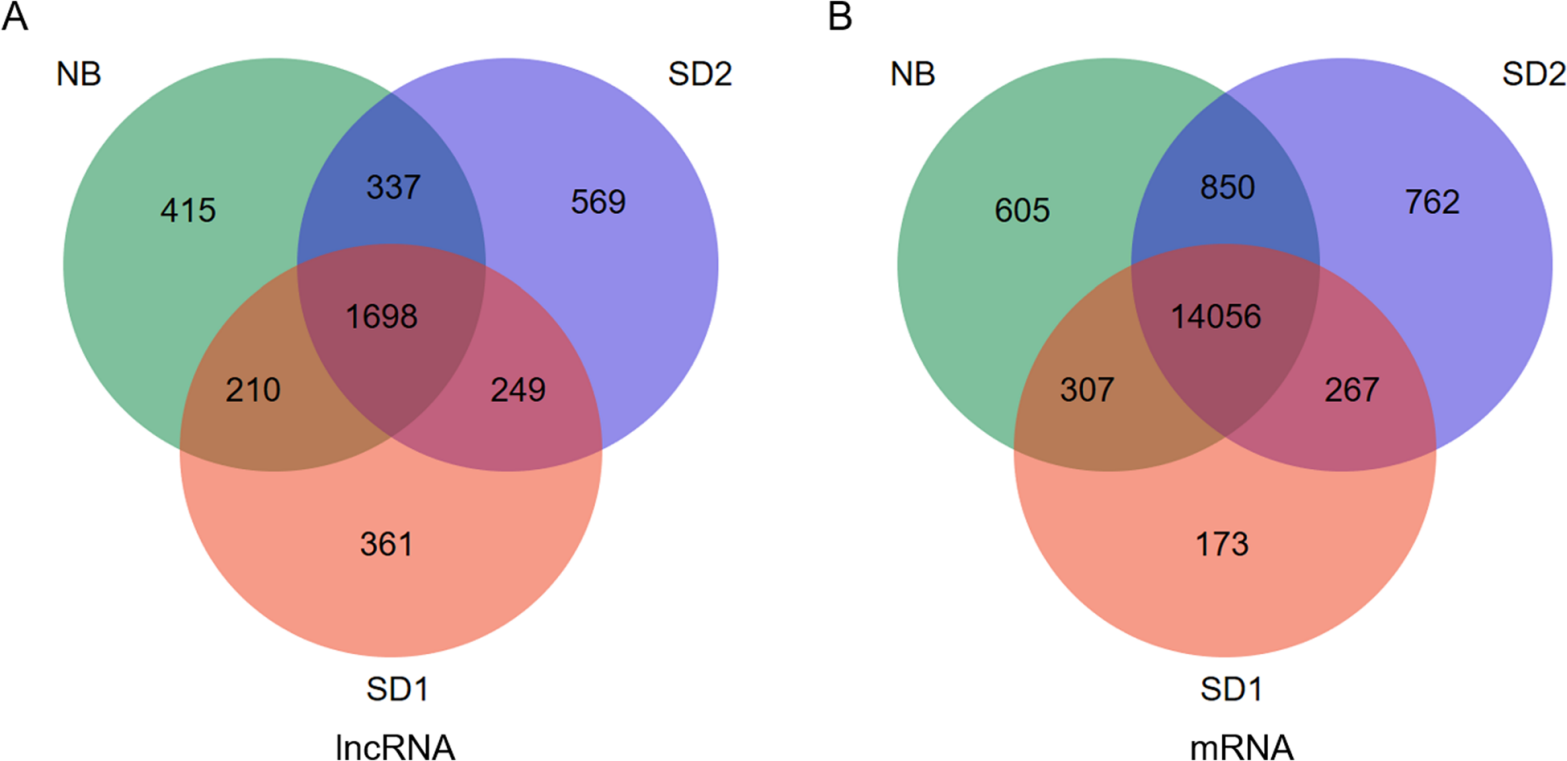
Venn diagrams showing the numbers of commonly and specifically expressed lncRNAs and mRNAs in different groups. **a** Numbers of commonly and specifically expressed lncRNAs in SD1, SD2 and NB based on a cutoff of FPKM> 0.1. **b** Numbers of commonly and specifically expressed mRNAs in SD1, SD2 and NB based on a cutoff of FPKM> 1. The FPKM value was denoted as the average value of three replicates

The relative gene expression level of each mRNA and lncRNA was normalized to Fragments Per Kilobase of transcript sequence per Millions base pairs sequenced (FPKM) value [42]. Based on the gene expression profiles, Spearman correlation analysis suggested that, three biological replicates were highly related with correlation efficiencies larger than 0.94 (*R*^2^ > 0.94), and were readily clustered into the same clade (Additional file 1: Fig. S1). Besides, SD1 and NB samples were clustered as neighboring clades, distant from SD2 clade (Fig. S1), indicating some transcriptional changes from SD1 to SD2 were recovered by NB. Statistics of mRNA and lncRNA expression profiles showed that lncRNAs were expressed at much lower levels compared with mRNAs (Fig. 2c). The open reading frame (ORF) lengths comparison results showed that, lncRNAs predominately harbored 100 bp-long ORFs, significantly shorter than that of mRNAs (Fig. 2d). Based on a criterion of average FPKM of three replicates> 0.1, we found 1698 lncRNAs were commonly expressed over different time points, and 361, 569 and 415 lncRNAs were specifically expressed at SD1, SD2, and NB, respectively (Fig. 3a). Meanwhile, based on the threshold of average FPKM of three replicates> 1, 14,056 mRNAs were detected across the whole time points (Fig. 3b). More genes (762) were specifically expressed at SD2 compared with the expressed genes at SD1 (173) and NB (605) (Fig. 3b). Coincidently, more lncRNAs and mRNAs were specifically expressed at SD2 than at NB, indicating SD may promote the expression of flowering genes but NB may repress this effect.

### Differential expression analysis of lncRNAs and mRNAs

DESeq2 [43] was employed to explore the differentially expressed lncRNAs (DE lncRNAs) and mRNAs (DEGs) between different groups with thresholds of FDR < 0.05 and |log2(fold change)| ≥ 1. In total, 91 DE lncRNAs and 1401 DEGs were obtained in comparisons of SD1_vs_SD2 and SD2_vs_NB (Table 2**,** Additional file 2: Table S1, Additional file 3: Table S2). Sixty-four lncRNAs were differentially expressed in SD1_vs_SD2, out of which 40 and 24 lncRNAs were up- and down-regulated in SD1_vs_SD2 (Table 2), respectively. After transferring from SD2 to NB, the expression levels of 20 and 31 lncRNAs were increased and decreased, respectively (Table 2). DEGs analysis showed that 693 and 309 genes were up- and down-regulated in SD1_vs_SD2, and 315 and 379 were up- and down-regulated in SD2_vs_NB, respectively (Table 2). These results indicated that many lncRNA and mRNA genes tended to be activated by SD whereas repressed by NB.

**Table 2:** Brief review of the differentially expressed lncRNAs and mRNA between different groups

Comparison	DE lncRNAs			DEGs		
	total	up	down	total	up	down
SD1_vs_SD2	64	40	24	1002	693	309
SD2_vs_NB	51	20	31	694	315	379

### Functional annotation of the differentially expressed lncRNAs in response to SD and NB

**Fig. 4:**
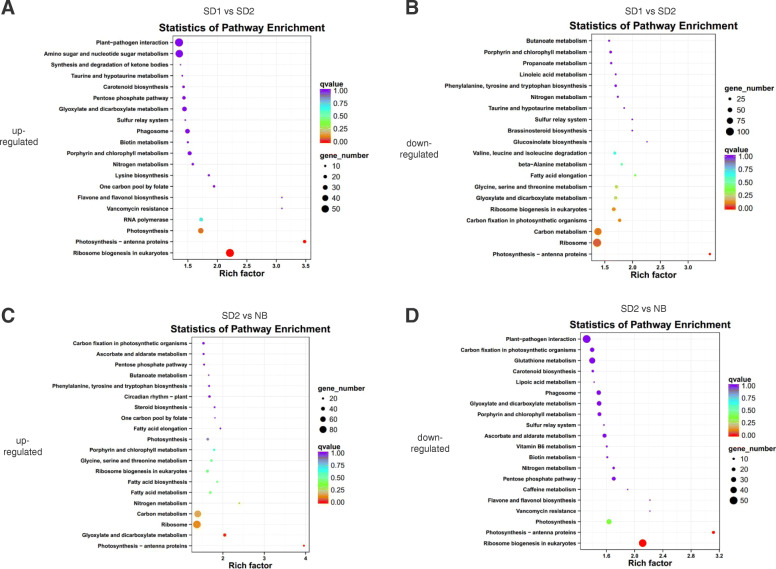
Functional annotation of the differentially expressed lncRNAs by Kyoto Encyclopedia of Genes and Genomes (KEGG) enrichment analysis. KEGG enrichment analysis of the up-regulated lncRNA-targets (**a**) and down-regulated lncRNA-targets (**b**) in comparison of SD1_vs_SD2. KEGG enrichment analysis of the up-regulated lncRNA-targets (**c**) and down-regulated lncRNA-targets (**d**) in comparison of SD2_vs_NB. Pathways with *P* value < 0.05 were considered as significantly enriched

To investigate the potential roles of DE lncRNAs, we performed functional annotation of the DE lncRNAs targets. The *cis* targets were selected from the mRNAs located at less than 100kbp downstream and upstream of each lncRNA. The *trans* targets were determined with cutoffs of Pearson’s correlation coefficient > 0.9 or < − 0.9 and *P* value < 0.01 between the expression profiles of mRNAs and lncRNAs. Co-expression and co-location analysis demonstrated that 5917 targets were regulated by the up-regulated lncRNAs in SD1_vs_SD2, including 29,401 lncRNA-mRNA pairs (Additional file 4: Table S3). Kyoto Encyclopedia of Genes and Genomes (KEGG) enrichment analysis indicated that these targets were abundant in pathways of “Ribosome biogenesis in eukaryotes” and “Photosynthesis-antenna proteins” (Fig. 4a). 5100 targets and 9340 lncRNA-mRNA pairs were predicted to be related with the down-regulated lncRNAs in SD1_vs_SD2 (Additional file 5: Table S4). “Photosynthesis-antenna proteins” was the only significantly enriched KEGG pathway for these target genes (Fig. 4b). In the network of the up-regulated lncRNAs in SD2_vs_NB, 4736 targets and 8420 lncRNA-mRNA pairs were contained (Additional file 6: Table S5). KEGG pathways including “Photosynthesis-antenna proteins” and “Glyoxylate and dicarboxylate metabolism” were highly related with these targets (Fig. 4c). 5929 targets and 20,438 lncRNA-mRNA pairs were involved in the network of the down-regulated lncRNAs in SD2_vs_NB (Additional file 7: Table S6). “Ribosome biogenesis in eukaryotes” and “Photosynthesis-antenna proteins” were the most significantly enriched KEGG pathways for these targets (Fig. 4d).

**Fig. 5:**
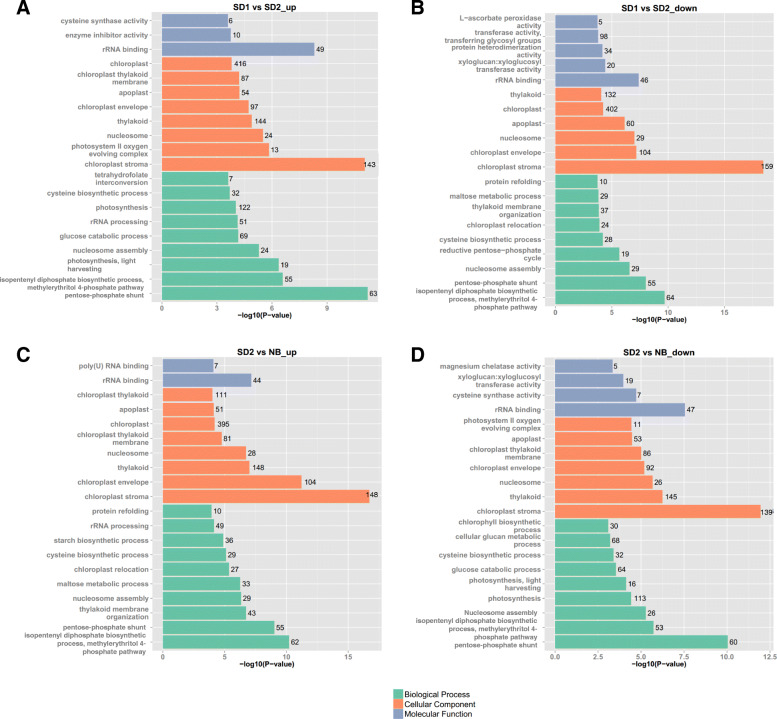
Functional annotation of the differentially expressed lncRNAs by Gene Ontology (GO) classification. GO classifications of the up-regulated lncRNA-targets (**a**) and down-regulated lncRNA-targets (**b**) in comparison of SD1_vs_SD2. GO classifications of the up-regulated lncRNA-targets (**c**) and down-regulated lncRNA-targets (**d**) in comparison of SD2_vs_NB. The top 20 significant GO terms in biological process, cellular component and molecular function categories are selected based on the cutoff of *P* value < 0.05. Number right-side each horizonal column indicates the number of genes classified in the GO term

In addition, to further reveal the roles of DE lncRNAs, we performed Gene Ontology (GO) enrichment analysis to obtain the top 20 significantly enriched GO terms of biological processes, cellular components and molecular functions. With regard to the targets of up-regulated lncRNAs under SD, the most significantly enriched GO terms for biological processes were “pentose-phosphate shunt”, “isopentenyl diphosphate biosynthetic process, methylerythritol 4-phosphate pathway”, photosynthesis, light harvesting”, “nucleosome assembly”, “glucose catabolic process”, “rRNA processing”, “photosynthesis”, “cysteine biosynthetic process” and “tetrahydrofolate interconversion” (Fig. 5a). The GO terms including “chloroplast stroma”, “photosystem II oxygen evolving complex”, “nucleosome”, “thylakoid”, “chloroplast envelope”, “apoplast”, “chloroplast thylakoid”, “membrane” and “chloroplast” were enriched in cellular component category (Fig. 5a). The GO terms of “rRNA binding”, “enzyme inhibitor activity” and “cysteine synthase activity” were highly represented in molecular functions (Fig. 5a). The targets of the down-regulated lncRNAs under SD were abundant in 9, 6 and 5 GO terms of biological process, cellular component, and molecular function categories, respectively. As far as biological processes were concerned, “isopentenyl diphosphate biosynthetic process, methylerythritol 4-phosphate pathway”, “pentose-phosphate shunt”, “nucleosome assembly”, “reductive pentose-phosphate cycle”, “cysteine biosynthetic process”, “chloroplast relocation”, “thylakoid membrane organization”, “maltose metabolic process”, and “protein refolding” were the most important GO terms (Fig. 5b). The 6 GO terms such as “chloroplast stroma”, “chloroplast envelope”, “nucleosome”, “apoplast”, “chloroplast” and “thylakoid” were highlighted in cellular components (Fig. 5b). The GO terms like “rRNA binding”, “xyloglucan: xyloglucosyl transferase activity”, “protein heterodimerization activity”, “transferase activity, transferring glycosyl groups” and “L-ascorbate peroxidase activity” were predominant in molecular function category (Fig. 5b).

GO enrichment analysis of the targets of up-regulated lncRNAs under NB showed that “isopentenyl diphosphate biosynthetic process, methylerythritol 4-phosphate pathway”, “pentose-phosphate shunt”, “thylakoid membrane organization”, “nucleosome assembly”, “maltose metabolic process”, “chloroplast relocation”, “cysteine biosynthetic process”, “starch biosynthetic process”, “rRNA processing” and “protein refolding” were highly represented in biological processes (Fig. 5c). GO terms “chloroplast stroma”, “chloroplast envelope”, “thylakoid”, “nucleosome”, “chloroplast thylakoid membrane”, “chloroplast”, “apoplast” and “chloroplast thylakoid” showed high representativeness of cellular components (Fig. 5c). With regard to molecular function category, targets were involved in “rRNA binding” and “poly (U) RNA binding” (Fig. 5c). The targets of down-regulated lncRNAs under NB were significantly enriched in 9, 7 and 4 GO terms of biological process, cellular component, and molecular function categories, respectively. The biological process GO terms contained “pentose-phosphate shunt”, “isopentenyl diphosphate biosynthetic process, methylerythritol 4-phosphate pathway”, “nucleosome assembly”, “photosynthesis”, “photosynthesis, light harvesting”, “glucose catabolic process”, “cysteine biosynthetic process”, “cellular glucan metabolic process” and “chlorophyll biosynthetic process” (Fig. 5d). With regard to cellular components, “chloroplast stroma”, “thylakoid”, “nucleosome”, “chloroplast envelope”, “chloroplast thylakoid membrane”, “apoplast” and “photosystem II oxygen evolving complex” were highly represented (Fig. 5d). As far as molecular functions were concerned, “rRNA binding”, “cysteine synthase activity”, “xyloglucan: xyloglucosyl transferase activity” and “magnesium chelatase activity” were significantly enriched (Fig. 5d).

### Prediction of the putative positive and negative flowering regulatory lncRNAs

**Fig. 6:**
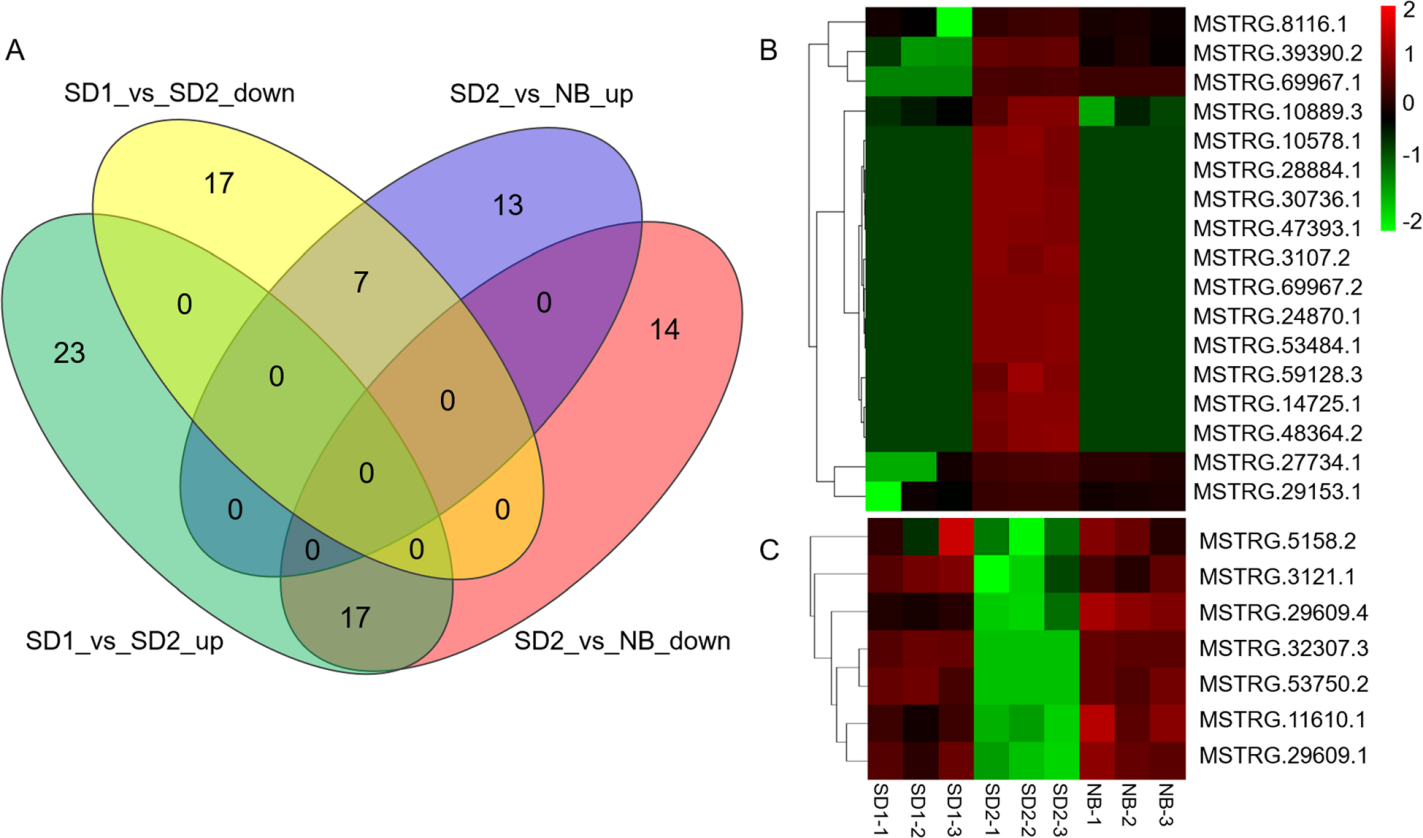
Identification and expression analysis of the putative positive and negative flowering lncRNAs. **a** Venn diagram showing the number of specific and common differentially expressed lncRNAs in comparisons of SD1_vs_SD2 and SD2_vs_NB. The 17 DE lncRNAs showing up-regulation in SD1_vs_SD2 but down-regulation in SD2_vs_NB were predicted to be putative positive flowering lncRNAs. The 7 DE lncRNAs showing down-regulation in SD1_vs_SD2 but up-regulation in SD2_vs_NB were predicted to be putative negative flowering lncRNAs. Heatmap showing the expression levels (log_10_FPKM) of putative positive (**b**) and negative (**c**) flowering lncRNAs in different samples

According to the flowering time illustrated above, SD and NB exerted positive and negative effects on quinoa flowering, respectively. To ascertain which lncRNAs were probably involved in quinoa flowering, we analyzed the transcriptional profiles of the 91 DE lncRNAs. Through venn diagram analysis, we found that most of the DE lncRNAs (67 out of 91) were solely affected by SD or NB, and no DE lncRNAs were constantly induced or repressed by both SD and NB (Fig. 6a). Interestingly, we noticed that the expression patterns of 24 DE lncRNAs displayed opposite trends under SD compared with NB (Fig. 6a). Based on the expression profiles, we inferred that the 17 SD-induced but NB-repressed lncRNAs were the putative positive flowering regulator (Fig. 6b). On the other hand, the 7 lncRNAs that repressed by SD but activated by NB were predicted to be the putative flowering repressors (Fig. 6c).

**Fig. 7:**
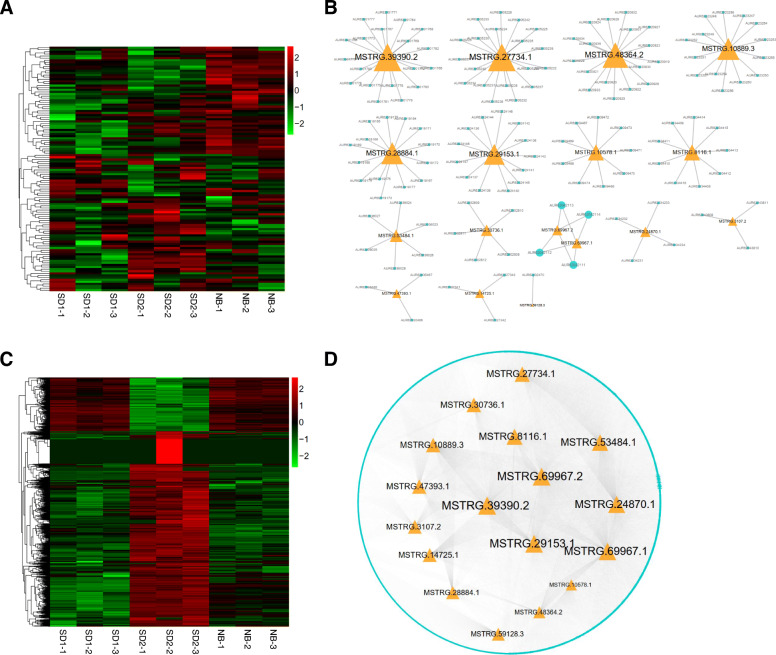
Expression and regulatory network analysis of the *cis* and *trans* targets of positive flowering lncRNAs. **a** Heatmap showing the expression levels (log_10_FPKM) of the *cis* targets in different samples. **b** Relationship between the positive flowering lncRNAs and their co-located *cis* targets. **c** Heatmap showing the expression levels (log_10_FPKM) of the *trans* targets in different samples. **d** Co-expression network analysis of the positive flowering lncRNAs and their *trans* targets. The orange triangles and light-blue circles stand for lncRNAs and mRNA targets, respectively. The node and font size reflect the degrees between lncRNAs and their targets

Further, to understand the functions of the lncRNAs, we investigated the regulatory networks of the putative flowering lncRNAs. With regard to the 17 positive flowering lncRNAs, 143 *cis* targets with 147 *cis-*acting lncRNA-mRNA pairs and 4034 *trans* targets with 16,844 *trans-*acting lncRNA-mRNA pairs were selected, respectively (Fig. 7) (Additional file 8: Table S7). *Cis*-acting network analysis indicated that the largest node *MSTRG.39390.2* co-located with 19 targets and *MSTRG.59128.3* only co-located with 1 target (Fig. 7a-b). Notably, a few important plant growth and development related transcription factors (TFs) were identified among the *cis* targets. For example, 3 *SCARECROW-like* genes of *GRAS* family (*AUR62001765*, *AUR62001766*, *AUR62001768*) co-located with *MSTRG.39390.2*; 1 *WOX* (*WUSCHEL*-*related homeobox*) (*AUR62035466*) co-located with *MSTRG.47393.1*. According to the *trans*-acting network of the positive flowering lncRNAs, the largest node *MSTRG.39390.2* and smallest node *MSTRG.10578.1* were predicted to regulate 1495 and 343 *trans* targets, respectively (Fig. 7c-d). Moreover, we found that 1 mRNA could be the common target of 1 to 14 lncRNAs and 423 mRNA genes were correlated with more than 10 lncRNAs (Fig. 7d). The expression heatmap demonstrated that most of these *trans* targets harbored the same expression trend with the lncRNAs (Fig. 7c). Functional annotation by KEGG enrichment analysis suggested that no pathways were significantly enriched for *cis* targets, whereas the *trans* target genes were highly abundant in pathways “Ribosome biogenesis in eukaryotes”, “Photosynthesis-antenna proteins” and “Photosynthesis” (Table 3).

**Table 3:** KEGG enrichment analysis of the *cis* and *trans* targets of the putative positive and negative flowering lncRNAs

	Targets	KEGG pathway	Pathway ID	Corrected ***P*** value
Positive flowering lncRNAs	*cis* targets	–	–	–
	*trans* targets	Ribosome biogenesis in eukaryotes	ko03008	6.19E-08
		Photosynthesis - antenna proteins	ko00196	6.43E-05
		Photosynthesis	ko00195	0.040877
Negative flowering lncRNAs	*cis* targets	Glutathione metabolism	ko00480	0.002922
	*trans* targets	Photosynthesis - antenna proteins	ko00196	6.30E-08
		Ribosome biogenesis in eukaryotes	ko03008	0.008546
		Porphyrin and chlorophyll metabolism	ko00860	0.016466
		Glyoxylate and dicarboxylate metabolism	ko00630	0.026896

**Fig. 8:**
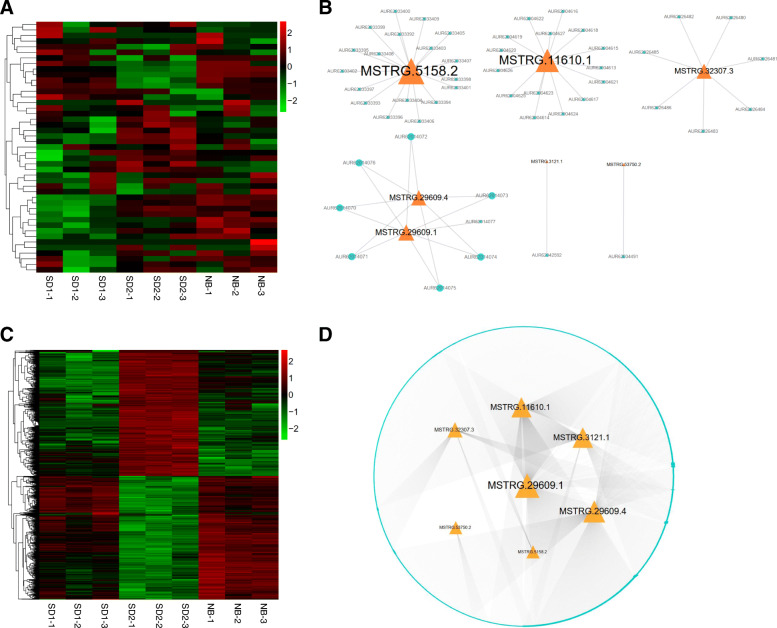
Expression and regulatory network analysis of the *cis* and *trans* targets of negative flowering lncRNAs. **a** Heatmap showing the expression levels (log_10_FPKM) of the *cis* targets in different samples. **b** Relationship between the negative flowering lncRNAs and their co-located *cis* targets. **c** Heatmap showing the expression levels (log_10_FPKM) of the *trans* targets in different samples. **d** Co-expression network analysis of the negative flowering lncRNAs and their *trans* targets. The orange triangles and light-blue circles stand for lncRNAs and mRNA targets, respectively. The node and font size reflect the degrees between lncRNAs and their targets

For the 7 putative flowering repressor lncRNAs, 50 *cis* targets with 57 *cis*-acting lncRNA-mRNA pairs and 2615 *trans* targets with 3985 *trans-*acting lncRNA-mRNA pairs may be correlated with these lncRNAs (Fig. 8) (Additional file 9: Table S8). From the *cis*-acting network we found that the largest node *MSTRG.5158.2* co-located with 18 targets, while the smallest nodes *MSTRG.53750.2* and *MSTRG.3121.1* only co-located with 1 target (Fig. 8a-b). With regard to the *trans* co-expression network, the largest node *MSTRG.29609.1* and the smallest node *MSTRG.5158.2* may interact with 1164 and 44 targets, respectively, and 1 mRNA could be correlated with 1 to 4 lncRNAs (Fig. 8c-d). The expression heatmap showed that about 50% of these *trans* targets harbored similar expression trend with the negative flowering lncRNAs (Fig. 8c). KEGG enrichment analysis indicated the *cis* targets were abundant in pathway “Glutathione metabolism”, and the *trans* targets were significantly enriched in pathways “Photosynthesis-antenna proteins”, “Ribosome biogenesis in eukaryotes”, “Porphyrin and chlorophyll metabolism” and “Glyoxylate and dicarboxylate metabolism” (Table 3).

### Co-expressed network associated with quinoa flowering

**Fig. 9:**
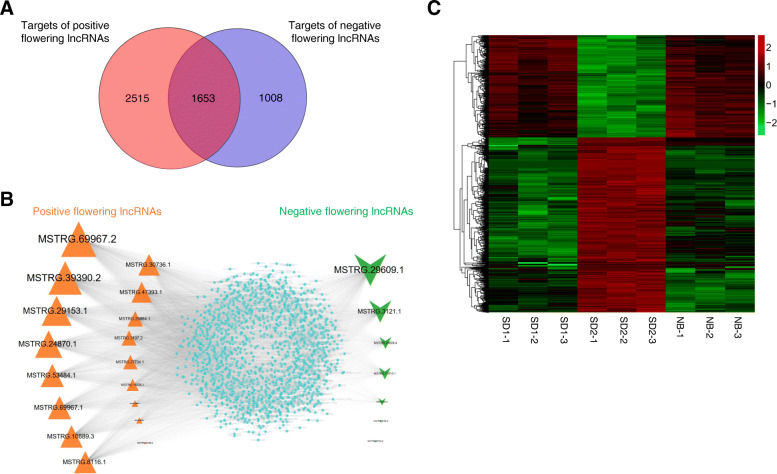
Identification of the common targets of positive and negative flowering lncRNAs. **a** Venn diagram showing the number of common and specific targets of positive and negative flowering lncRNAs. **b** Co-expression network of 17 positive, 7 negative flowering lncRNAs and their common targets. The orange triangles, green V and light-blue circles represent positive, negative flowering lncRNAs and their common targets, respectively. The node and font size reflect the degrees between lncRNAs and their targets. **c** Heatmap showing the expression levels (log_10_FPKM) of the 1653 common targets in different samples

**Fig. 10:**
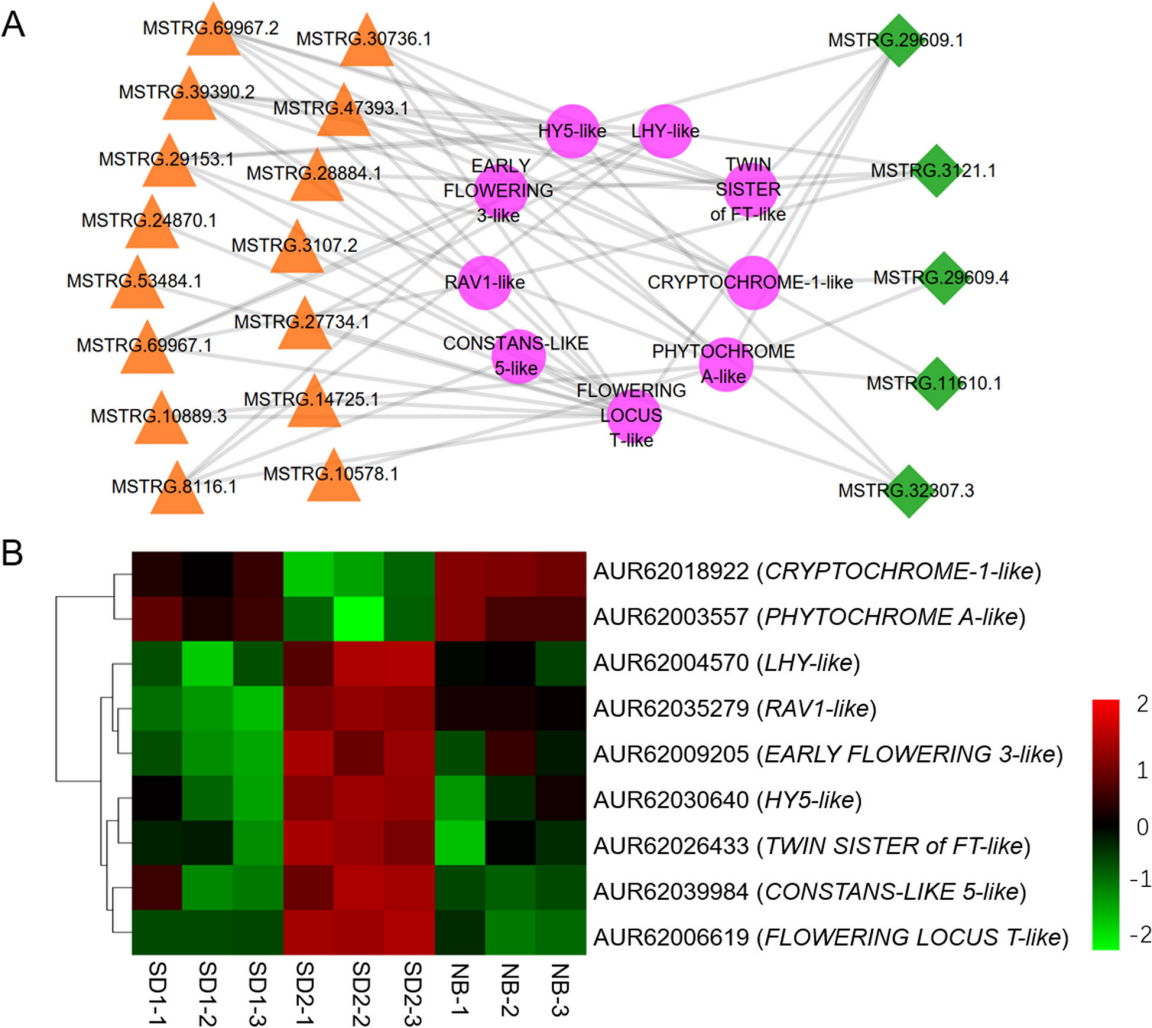
Construction of the core network involved in quinoa flowering. **a** Co-expression network of the positive, negative flowering lncRNAs and vital flowering homologs. The orange triangles, green rhombuses and purple circles represent positive, negative flowering lncRNAs and the vital flowering homologs, respectively. **b** Heatmap showing the expression levels (log_10_FPKM) of the vital flowering homologs in different samples

Furthermore, by comparing the seed nodes in the positive and negative lncRNAs flowering networks, we noticed that 1653 mRNAs were simultaneously regulated by both types of regulatory lncRNAs, accounting for 39.66% (1653/4168) and 62.12% (1653/2661) of the positive and negative network targets, respectively (Fig. 9a, b). The expression heatmap indicated that most of these targets harbor higher expression levels under SD than under NB (Fig. 9c). Co-expression network analysis demonstrated that the largest node *MSTRG.69967.2* was linked with 1176 targets while *MSTRG.59128.3* was only co-expressed with 1 target, and the top-5-degree target nodes (*Chenopodium_quinoa_newGene_17423*, *AUR62040917*, *AUR62018533*, *AUR62015897*, *AUR62000858*) were regulated by as many as 17 lncRNAs (Fig. 9b). Intriguingly, among the 1653 targets, we found that a few vital plant growth and development related modulators co-expressed with 15 positive and 5 negative putative flowering lncRNAs (Fig. 10a-b). For example, the *FLOWERING LOCUS T-like* (*FT-like*) homolog (*AUR62006619*), which belongs to the *PHOSPHATIDYLETHANOLAMINE-BINDING PROTEIN* (*PEBP*) family and functions as a crucial floral integrator triggering downstream floral organogenesis in plants [10], was predicted to be co-expressed with 13 positive (*MSTRG.53484.1*, *MSTRG.30736.1*, *MSTRG.39390.2*, *MSTRG.27734.1*, *MSTRG.14725.1*, *MSTRG.47393.1*, *MSTRG.3107.2*, *MSTRG.29153.1*, *MSTRG.24870.1*, *MSTRG.10889.3*, *MSTRG.8116.1*, *MSTRG.69967.1*, *MSTRG.69967.2*) and 1 negative (*MSTRG.29609.1*) flowering lncRNAs (Fig. 10a-b). Another *PEBP* member *TWIN SISTER of FT-like* (*TSF-like*) homolog (*AUR62026433*), which shares similar functions with *FT*, may be regulated by 5 positive (*MSTRG.30736.1*, *MSTRG.39390.2*, *MSTRG.47393.1*, *MSTRG.28884.1*, *MSTRG.69967.2*) and 2 negative (*MSTRG.3121.1*, *MSTRG.29609.1*) flowering lncRNAs (Fig. 10a-b). *AUR62039984*, encoding a B-BOX Zinc Finger protein *CONSTANS-like* (*CO-like*) which acts upstream of *FT* in photoperiod-dependent manner [44], was tightly correlated with the flowering activator *MSTRG.8116.1* and the flowering repressor *MSTRG.32307.3*. *AUR62009205*, an *EARLY FLOWERING 3-like* homolog which plays important roles in maintaining circadian rhythms and controlling flowering time [45, 46], was the *trans* target of 2 positive (*MSTRG.32307.3*, *MSTRG.3121.1*) and 2 negative (*MSTRG.39390.2*, *MSTRG.69967.1*) flowering lncRNAs (Fig. 10a-b). The *MYB*-type *LATE ELONGATED HYPOCOTYL* (*LHY*) is a core component of circadian clock controlling many clock outputs such as flowering and photomorphogenesis [47]. In this study, we found that the quinoa *LHY* homolog (*AUR62004570*) was co-expressed with 6 lncRNAs (5 positive lncRNAs: *MSTRG.39390.2*, *MSTRG.27734.1*, *MSTRG.29153.1*, *MSTRG.8116.1*, *MSTRG.69967.1*; 1 negative lncRNA: *MSTRG.3121.1*) (Fig. 10a-b). The bZIP transcription factor *ELONGATED HYPOCOTYL 5* (*HY5*) mediates blue light signaling to modulate circadian clock rhythms and plays multifaceted roles in plant growth and development [48]. The quinoa *HY5* homolog *AUR62030640* was correlated with 4 positive (*MSTRG.39390.2*, *MSTRG.29153.1*, *MSTRG.8116.1*, *MSTRG.69967.2*) and 2 negative lncRNAs (*MSTRG.32307.3*, *MSTRG.29609.1*) (Fig. 10a-b). Previous studies suggested that the *RELATED TO ABI3/VP1* (*RAV*) family members delay heading time by repressing *Heading date 3a* (*Hd3a*) and *GIBBERELLIN 3-OXIDASE1/2* in rice (*Oryza sativa*) [49]. In the present study, we found that the quinoa *RAV1* homolog (*AUR62035279*) was regulated by 3 lncRNAs (2 positive lncRNAs, *MSTRG.39390.2*, *MSTRG.69967.1*; 1 negative lncRNA: *MSTRG.3121.1*) (Fig. 10a-b). Numerous evidences have indicated that photoreceptor *PHYTOCHROME A* (*PHYA*) mainly mediates light responses to various ratios of red/far-red light. In rice, the *phyA* mutation in *phyB* or *phyC* background leads to dramatic early heading [14]. The quinoa *PHYA* homolog (*AUR62003557*) was identified as the target of 8 lncRNAs (5 positive lncRNAs, *MSTRG.30736.1*, *MSTRG.29153.1*, *MSTRG.10889.3*, *MSTRG.10578.1*, *MSTRG.69967.2*; 3 negative lncRNAs: *MSTRG.11610.1*, *MSTRG.29609.4*, *MSTRG.29609.1*) (Fig. 10a-b). In addition, we found another photoreceptor, the blue light photoreceptor *CRYPTOCHROME1* homolog (*AUR62018922*) which is involved in entrainment of circadian clock and photoperiodic flowering [50], was linked with 7 lncRNAs (4 positive lncRNAs, *MSTRG.30736.1*, *MSTRG.47393.1*, *MSTRG.28884.1*, *MSTRG.69967.2*; 3 negative lncRNAs: *MSTRG.11610.1*, *MSTRG.29609.4*, *MSTRG.29609.1*) (Fig. 10a-b). Consequently, the tight association with these photoreceptors, circadian clock and florigen genes indicates these lncRNAs may play important roles in photoperiodic flowering of quinoa.

## Discussion

Flowering is a vital process closely associated with the final yield of crops. Previous studies have revealed the roles of many protein-coding genes in flowering time regulation. However, knowledge about the functions of lncRNAs in plant flowering and yield regulation is still limited. Until recently, a few studies have deciphered the pivotal roles of lncRNAs in flowering and yield regulation. In Arabidopsis, lncRNAs *COLDAIR* and *COOLAIR* were proven to regulate vernalization and flowering time via epigenic-repression of *FLC* [23, 24]. In rice, *EARLY FLOWERING-COMPLETELY DOMINANT* (*EF-CD*) encodes an antisense lncRNA overlapping with the floral activator *OsSOC1* to shorten maturation period without concomitant yield penalty [51]. Mining of specific lncRNAs may provide novel strategies for crop yield improvement.

Quinoa is an annual-flowering crop originated from the low latitude Andeans region. The flowering time and yield of some quinoa cultivars tend to vary when introduced into different countries [52]. However, the flowering regulators, especially the lncRNAs mediated flowering networks, remain to be revealed. SD combined with NB approach is an effective way for exploring the floral regulators. In this study, we found NB repressed the quinoa floral buds development in contrast to the flowering-promoting effects of SD (Fig. 1a-d). These results were consistent with the observations in other SDPs such as rice [11] and soybean [12], indicating the conserved flowering-repressing effects of NB on SDPs. To uncover the specific lncRNAs involved in NB responses, we compared the transcriptome differences between plants grown under SD and NB. By high-throughput sequencing, we identified 4914 lncRNAs from quinoa leaf tissues, less than the number of lncRNAs identified from tomato (*Solanum lycopersicum*) [53] but more than that identified from *Cucumis melo* [31], Cassava (*Manihot esculenta*) [54] and peanut (*Arachis hypogaea*) [55]. Meanwhile, only 605 (12.3%) shared similar sequences with the known lncRNAs in the NONCODE database. We presumed that the lncRNAs number and sequence conservation may be highly associated with tissue specificity and plant species. In this study, we identified 91 DE lncRNAs, most of which (73.6%) were affected solely either by SD or NB. Considering the contrary effects of SD and NB, we neglected this part of DE lncRNAs, and speculated that 17 and 7 lncRNAs, affected by both SD and NB in antagonizing manner, were the putative positive and negative flowering regulators, respectively (Fig. 6a-c).

To investigate the roles of the lncRNAs, we performed KEGG functional enrichment analysis of the *cis* and *trans* targets of the putative flowering lncRNAs. Pathway “Glutathione metabolism” was specific for the *cis* targets of negative flowering lncRNAs. “Photosynthesis” was the specific pathway for the *trans* targets of positive flowering lncRNAs, while “Porphyrin and chlorophyll metabolism” and “Glyoxylate and dicarboxylate metabolism” were specific for the *trans* targets of negative flowering lncRNAs (Table 3). These specific pathways may provide valuable cues for investigating the roles of these flowering lncRNAs in future.

Comparison of the *trans* targets of positive and negative flowering lncRNAs revealed that a considerable number of targets (1653 genes) co-expressed with both types of lncRNAs (Fig. 9a), indicative of competing flowering regulatory networks in quinoa. As supporting evidences, annotation of the common targets revealed a few floral homologs which may function as vital mediators of the antagonizing lncRNAs. Flowering time is largely influenced by endogenous circadian clock and external light conditions which are output to the final integrators *FT* in leaves [4]. As discovered in a lot of plant species, there are multiple *FT* homologs with highly conserved sequences in their genomes. However, different *FT* homologs may experience functional diversification because of the varied expression patterns [56]. For example, in rice there are 11 *FT* homologous genes, among which both *Hd3a* and *RFT1* are floral activators [11, 57]. However, only *Hd3a* showed obvious expression suppression by NB [11], which is the principal cause of the NB effect in rice. Ten *FT* homologs are isolated in soybean genome, yet, according to the expression pattern they are divided into SD-, LD-specific and photoperiod-independent groups [58]. Functional characterization suggests that *GmFT1a* and *GmFT2a*/*5a* have opposite effects in flowering of soybean [58]. In *Chenopodium rubrum* (*C. rubrum*), a close relative of quinoa, *CrFTL1* is SD-inducible and inhibited by NB, whereas *CrFTL2* is constitutively expressed [59]. In allotetraploid quinoa, 11 putative *FT* homologs have been identified [60]. However, which are NB-responsive is not determined. In this study, we found that only *AUR62006619* (*FT-like*) and *AUR62026433* (*TSF-like*) were significantly affected by NB (Fig. 10**)**. *AUR62006619* and *AUR62026433* were down-regulated by 8-fold and 2-fold, respectively, under NB than under SD. Thus, we speculated that these two *FT* homologs are probably the essential mediators for NB responses in quinoa. Co-expression network analysis showed that the quinoa *FT-like* homolog (*AUR62006619*) and *TSF-like* homolog (*AUR62026433*) were regulated by 14 and 7 lncRNAs, respectively. Noteworthy, both of these two quinoa *FT* homologs were commonly regulated by 5 lncRNAs (4 positive lncRNAs: *MSTRG.30736.1*, *MSTRG.39390.2*, *MSTRG.47393.1*, *MSTRG.69967.2*; 1 negative lncRNAs: *MSTRG.29609.1*) (Fig. 10**)**, suggesting these two homologs may function through partially overlapped signal cascade.

The B-BOX type *CO*, a direct activator of *FT*, functions as the output of circadian clock [61]. In rice, the transcriptional level of *CO* homolog-*HD1* is not obviously changed by NB [11]. However, different scenarios are observed in other plants. For instance, in Chrysanthemum (*Chrysanthemum morifolium*), the expression of *CmCOL1* is slightly reduced by NB [62]. In *C. rubrum*, two *CrCOL*s genes were down-regulated by dark-light transition [59]. Similarly, we found that 1 out 6 *CO-like* homologs (*AUR62039984*) in quinoa was down-regulated by NB. These results indicate the specific roles of *CO* homologs of different plants in NB responses. Coincidently, three circadian clock related genes, *LHY-like* (*AUR62004570*), *ELF3-like* (*AUR62009205*) and *HY5-like* (*AUR62030640*), were also repressed by NB. In addition, *LHY-like*, *HY5-like* and *CO-like* were commonly regulated by one lncRNA (*MSTRG.8116.1*) (Fig. 10**)**. These results imply the possible positive roles of these circadian clock genes on quinoa *CO-like*. Photoreceptors are essential for perceiving light signals to regulate circadian clock and flowering time. Phytochromes and Cryptochromes are responsible for red/far-red and blue light perceiving, respectively. In quinoa, there are 6 Phytochromes (2 *PHYA*, 2 *PHYB* and 2 *PHYC*) and 4 Cryptochromes (2 *CRY1* and 2 *CRY2*) [60]. In this study, we found 1 *PHYA* and 1 *CRY1* were specifically up-regulated by NB and were commonly co-expressed with as many as 5 lncRNAs (*MSTRG.69967.2*, *MSTRG.30736.1*, *MSTRG.29609.1*, *MSTRG.29609.4*, *MSTRG.11610.1* and *MSTRG.30736.1*) (Fig. 10**)**. It is of note that *MSTRG.69967.2*, *MSTRG.30736.1* and *MSTRG.29609.1* also participated in regulating the two *FT* homologs (Fig. 10**)**. Thus, these data suggested *PHYA* and *CRY1* may regulate flowering time by controlling the two *FT* homologs. Together, these putative flowering lncRNAs may mediate NB responses by regulating the key flowering genes. We believe further functional investigation on these lncRNAs will shed light on their roles in quinoa flowering.

## Conclusion

In summary, in this study we performed SS RNA-seq to explore genome-wide lncRNAs in quinoa. By analyzing the transcriptome data under SD and NB, we predict the specific lncRNAs that may be required for photoperiodic flowering. To our knowledge, this is the first systematic investigation on the lncRNAs involved in NB-responses of quinoa. In total, we identified 17 putative positive- and 7 putative negative-flowering lncRNAs. Co-expression network analysis indicated that 15 positive-flowering lncRNAs may compete with 5 negative-flowering lncRNAs to control a few pivotal flowering homologous genes, which might be the basis of NB responses in quinoa. Collectively, these results will deepen our understanding of the roles of lncRNAs in flowering, and provide valuable genetic resources for yield improvement of quinoa.

## Methods

### Plant materials and growth conditions

Quinoa (*Chenopodium quinoa*) cultivar “HL1” and “HL2” were used for flowering time analysis. The two cultivars have nearly the same floral transition time, however, “HL2” needs longer time (about 115 d) compared with “HL1” (about 60 d) for post-flowering development. Seeds were harvested from the Experimental Station of Key Laboratory of Coarse Cereal Processing, Ministry of Agriculture and Rural Affairs, in 2019 at Liangshan, Sichuan province. Seeds were sown in a pot with 20 cm in diameter and 18 cm in depth. Quinoa seedlings were maintained for 14 d in a greenhouse at Chengdu University under natural day conditions. Then, the seedlings pots were transferred into growth chambers (Jiang Nan, Ning Bo, China) subjecting to SD and NB treatment. The floral buds developmental situation of both cultivers under different conditions was observed every day.

For RNA-seq, the seeds of “HL1” were sown in pots with the same size as described above. After sowing, the pots were placed in growth chambers for SD treatment with daily cycles of 8 h light at 25 °C and 16 h dark at 23 °C. The relative humidity was 70%, and light intensity was 750 μmol.m^− 2^.s^− 1^. On 7 DAS, 15 robust shoots were retained in each pot by removing the spared ones. On 14 DAS (SD1) and 26 DAS (SD2), the top two fully expanded leaves of each seedling were collected at 2 h after dawn, respectively. Then, the growth conditions were reset to night-break conditions (NB) with daily cycles of 8 h light at 25 °C and 7 h dark, 1 h light exposure and 8 h dark at 23 °C. After 2 d NB treatment, namely on 28 DAS, the leaf samples were collected as above. Sampling was performed with three replicates and each replicate contains 5 individual plants. The harvested fresh leaf samples were immediately frozen by liquid nitrogen and stored at − 80 °C before RNA extraction.

### Construction of strand-specific RNA sequencing libraries

Total RNA was extracted from each leaves sample using Trizol reagent (Invitrogen, CA, USA) according to the manufacturer’s instructions. RNA degradation was checked by 1.5% agarose gel electrophoresis. RNA concentration and purity of each sample were measured to be qualified for downstream experiments. Only the samples with RIN (RNA Integrity Number) value larger than 7.5 were used for long-noncoding RNA sequencing libraries construction. An amount of 1.5 μg total RNA per sample was input for rRNA removal reaction by using the Ribo-Zero rRNA Removal Kit (Epicentre, Madison, WI, USA). Then, strand-specific RNA sequencing libraries were generated using NEBNext^R^ Ultra™ Directional RNA Library Prep Kit for Illumina^R^ (NEB, USA) according to manufacturer’s protocol.

### Data analysis

RNA sequencing was performed on an Illumina platform (Hiseq 4000) to generate 150 bp paired-end reads. Raw reads from 9 libraries were filtered by removing the unqualified reads (adapter-, ploy-N-containing and low-quality reads) to obtain clean reads. By applying HISAT2 software [38], clean reads were mapped to the quinoa reference genome sequences (https://www.cbrc.kaust.edu.sa/chenopodiumdb) [37]. Based on the mapping results, StringTie [39] was used to re-construct the transcripts. Gffcompare program [40] was used to annotate the assembled transcripts, and the unannotated transcripts were further used for long-noncoding RNA prediction. Transcripts with lengths > 200 bp and exon number ≥ 2 were retained as lncRNA candidates whose protein-coding potential were further evaluated by using CPC (Coding Potential Calculator) [63], CNCI (Coding-Non-Coding Index) [64], CPAT (Coding Potential Assessment Tool) [65] and Pfam programs [66] to obtain the final lncRNAs. LncRNAs were categorized into different types of lncRNAs including lincRNA, intronic lncRNA, anti-sense lncRNA and sense lncRNA by using Cuffcompare program.

### Differential expression analysis

The expression levels of both mRNA and lncRNA genes were calculated and normalized to FPKM (fragments per kilo-base of exon per million fragments mapped) [42] value using StringTie [39]. Differentially expressed genes (DEGs) between different groups were determined by using DESeq2 R package [43] based on the negative binomial distribution model. The resulting *P* values were adjusted using the Benjamini and Hochberg’s approach for controlling the false discovery rate (FDR). Thresholds of an FDR < 0.05 and |log2 (fold change)| ≥ 1 were applied to call DEGs.

### Target gene prediction

For *cis* targets identification, the protein-coding genes located at 100 kb upstream and downstream of lncRNA were selected and functionally annotated. To identify the *trans* targets, the correlation between mRNAs and lncRNAs was evaluated using the Pearson’s correlation coefficient (PCC) based on their expression profiles at different samples. mRNA-lncRNA pairs with |PCC value| > 0.9 and *P* value < 0.01 were designated as co-expressed gene pairs in which the mRNA genes were identified as the *trans* targets of lncRNAs.

### Functional enrichment analysis of the targets of differentially expressed lncRNAs

To understand the biological function of differentially expressed lncRNAs (DE lncRNAs), Kyoto Encyclopedia of Genes and Genomes (KEGG) [67] and Gene Ontology enrichment analysis [68] of the corresponding target genes was conducted. KEGG enrichment analysis was performed by using KOBAS software and the pathways with corrected *P* value less than 0.05 were taken as significantly enriched by the target genes. GO enrichment analysis of the target genes was implemented by the topGO R packages. The most significantly enriched GO terms were selected with the cutoff of corrected *P* value < 0.05.

### Co-expression network analysis and visualization

Based on the expression data, the PCC between lncRNAs and mRNAs was obtained. Co-expressed lncRNA-mRNA pairs were visualized using Cytoscape (Version 3.7.2) [69]. By using the Cytoscape tools “Network Analyzer-Network analysis”, the network was analyzed and the degrees of nodes were obtained.

## Supplementary Information

**Additional file 1: Fig. S1.** Spearman correlation coefficients between different samples

**Additional file 2: Table S1.** Expression levels of all DE lncRNAs

**Additional file 3: Table S2.** Expression levels of all DEGs

**Additional file 4: Table S3.** Targets of the up-regulated lncRNAs in SD1_vs_SD2

**Additional file 5: Table S4.** Targets of the down-regulated lncRNAs in SD1_vs_SD2

**Additional file 6: Table S5.** Targets of the up-regulated lncRNAs in SD2_vs_NB

**Additional file 7: Table S6.** Targets of the down-regulated lncRNAs in SD2_vs_NB

**Additional file 8: Table S7.** Network of the 17 positive flowering lncRNAs

**Additional file 9: Table S8.** Network of the 7 negative flowering lncRNAs

## Data Availability

The transcriptome raw data produced in this study is deposited at NCBI SRA database (http://trace.ncbi.nlm.nih.gov/Traces/sra) under accession PRJNA673052.

## Abbreviations

SD: Short-day
NB: Night-break
lncRNAs: Long noncoding RNAs
FPKM: Fragments Per Kilobase of transcript sequence per Millions base pairs sequenced
SDP: Short-day plant
LDP: Long-day plant
DAS: Days after sowing
GO: Gene ontology
KEGG: Kyoto Encyclopedia of Genes and Genomes
PCC: Pearson’s correlation coefficient
CPC: Coding Potential Calculator
CNCI: Coding-Non-Coding Index
CPAT: Coding Potential Assessment Tool
