# Supplementary material for: Identification of the specific long-noncoding RNAs involved in night-break mediated flowering retardation in Chenopodium quinoa
Source: BMC Genomics. 2021 Apr 19;22:284. doi: 10.1186/s12864-021-07605-2 (PMC8056640; doi:10.1186/s12864-021-07605-2)
Supplement: Supplementary file 1 — Additional file 1: Fig. S1. Spearman correlation coefficients between different samples [file 12864_2021_7605_MOESM1_ESM.docx]

**Supplementary materials**


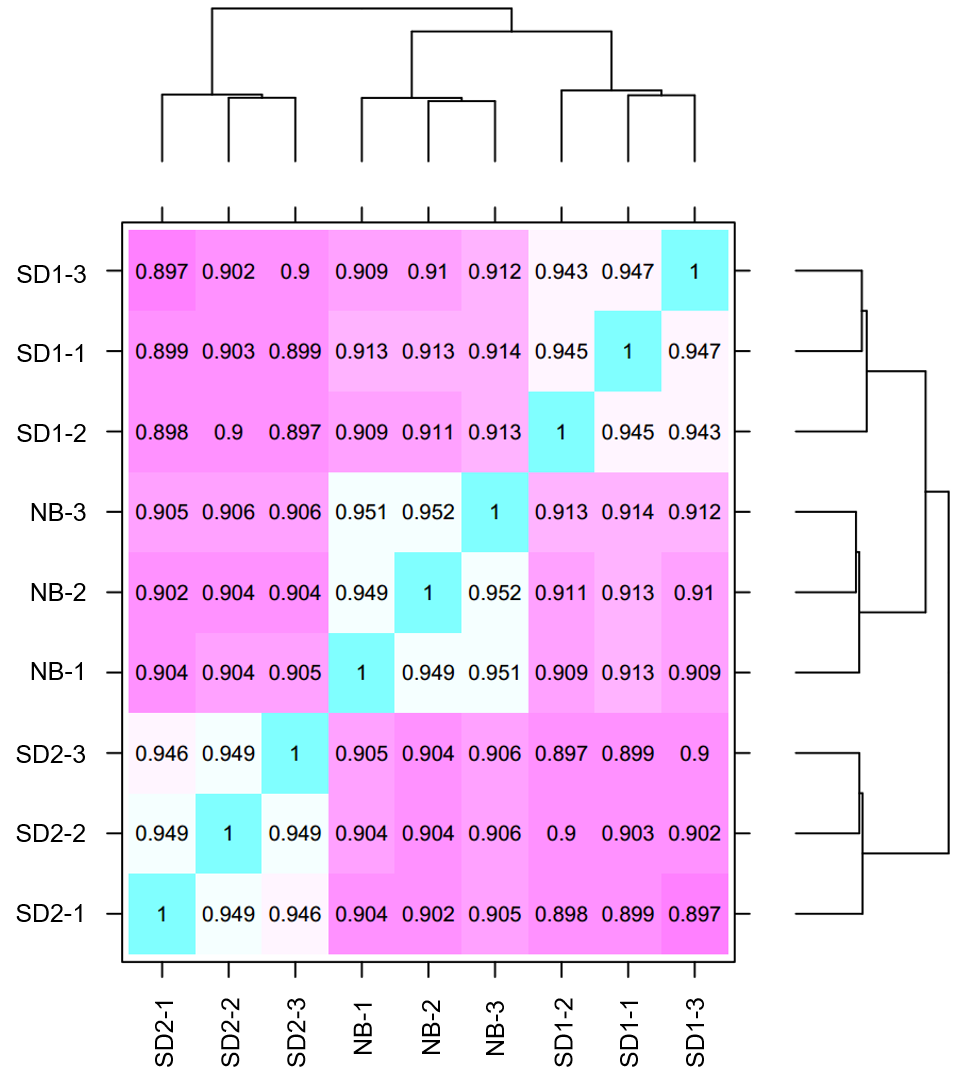


Fig. S1. Spearman correlation coefficients between different samples.

Additional files

Additional file 1: Fig. S1. Spearman correlation coefficients between different samples.

Additional file 2: Table S1. Expression levels of all DE lncRNAs.

Additional file 3: Table S2. Expression levels of all DEGs.

Additional file 4: Table S3. Targets of the up-regulated lncRNAs in SD1_vs_SD2.

Additional file 5: Table S4. Targets of the down-regulated lncRNAs in SD1_vs_SD2.

Additional file 6: Table S5. Targets of the up-regulated lncRNAs in SD2_vs_NB.

Additional file 7: Table S6. Targets of the down-regulated lncRNAs in SD2_vs_NB.

Additional file 8: Table S7. Network of the 17 positive flowering lncRNAs.

Additional file 9: Table S8. Network of the 7 negative flowering lncRNAs.
